# Children's Country Holidays Fund

**Published:** 1889-08-17

**Authors:** 


### Children's Country Holidays Fund.

### THE POOREST CHILDREN.

How far it is possible to send the poorest children away into the country is often warmly debated. At the outset we must clearly understand who these poorest children are. Most people would at once think of the shoeless dirty girl of the slums, or the ragged boy who turns cartwheels by the side of an omnibus. But it is very questionable if this class is the poorest. Cartwheels pay better at the time than a regular attendance at school, and many a match seller refuses regular work because the earnings of street selling are so large—given the proper show of rags and poverty to draw out the purse.

Broadly speaking, these children cannot be sent for the country holiday. First of all, the genuine street arab would not go except in his own way. Next, his language and habits make him a hardly desirable importation for a country village. Further, a very large number of this class (essentially idle, and holiday making all the year round) get their country change on tramp, or in the fruit and hop gardens of Kent and Sussex. But yet if children are sent tidy after much hard work of the mothers, or with clothes given by the committee, or lent for the occasion by a brother or neighbour, the public call out that the poorest children are not taken. It is so old a truth that you cannot judge poverty or anything else by appearances that it is extraordinary how many people forget it, and expect to see the parties of holiday children all dirty, unshod, and in rags. As it is, the country people are always complaining of personal want of cleanliness, of horrible undergarments, and the total absence of any change of clothing. Every year scores of country centres are closed because the country people will not have their comfort so interfered with.

It is just possible that a lower stratum might be touched if some self-sacrificing gentleman would volunteer to personally conduct a few boys to some camp or hoppers' huts; but very few would do this, and fewer still repeat the experiment.

It is certain that decent country cottagers will not put up with any lower class than they already take. A few days ago one lady wrote from her local committee to the secretary: "I shall be most glad if you will send me some clothes, for we have to bath and clothe many children before they can be sent away"; while another lady wrote last week: "I have this afternoon heard that one little fellow is temporarily in such rags he is not able to appear because all his clothes are washed ready for the country."

Now, in spite of all this undoubted poverty, the parents pay on an average 2s. a week to the Children's Country Holidays Fund, and thereby they enabled between 5,000 and 6,000 more children to go last year. Many save weeks, and even months before, and in some cases the children have done little bits of work for the visitor to add a few pence to their contribution. The truth is that it is hardly possible to keep a child on less than 3d. a day, and by careful management and by paying small sums at a time the very poorest can at once help themselves and others. On the whole it may fairly be claimed that the Children's Country Holidays Fund sends the very poor, though with them is a sprinkling of artisans' children.

One word of warning may perhaps be given; some local committees try to fix a minimum sum required: it is, we think, unwise to have any unelastic rule of this sort. The regulations of the council say that parents must contribute in proportion to their means—it would surely be a great mistake to insist on any uniformity. It would certainly avoid all the trouble of enquiry into means, but at the same time do away with the exercise of the kindly personal influence, which is the real compelling power. It would be exceedingly unfortunate if the poorer should feel that they were being forced up to a level which they could hardly afford, while those in better circumstances were allowed to pay less than they could and would if asked.

